# Assessing the impact of an educational intervention program based on the theory of planned behavior on the nutritional behaviors of adolescents and young adults with PCOS in Iran: a field trial study

**DOI:** 10.1186/s12887-021-02784-z

**Published:** 2021-07-14

**Authors:** Leila Hajivandi, Mahnaz Noroozi, Firoozeh Mostafavi, Maryam Ekramzadeh

**Affiliations:** 1 Department of Nursing and Midwifery, Kazerun Medical Sciences Branch, Islamic Azad University, Kazerun, Iran; 2 Department of Midwifery and Reproductive Health, School of Nursing and Midwifery, Isfahan University of Medical Sciences, Isfahan, Iran; 3 Department of Health Education and Promotion, School of Health, Isfahan University of Medical Sciences, Isfahan, Iran; 4 Nutrition Research Center, Department of Clinical Nutrition, School of Nutrition and Food Sciences, Shiraz University of Medical Sciences, Shiraz, Iran

**Keywords:** Polycystic ovary syndrome, Adolescent, Theory of planned behavior, Iran

## Abstract

**Background:**

Polycystic ovary syndrome is the most common endocrine disorder among adolescents and adults. Given the importance of healthy nutritional behaviors in management of this disease, the present study was conducted to determine the impact of an educational intervention program based on the theory of planned behavior on the nutritional behaviors of adolescents and young adults with polycystic ovary syndrome.

**Methods:**

In this field trial study, 72 participants aged between 15 and 21 years old from four gynecology clinics in Shiraz, Iran, were assigned into the intervention and control groups. Educational intervention program was implemented based on the theory of planned behavior over 4 sessions during two weeks. The data collection tools were researcher-made questionnaires of demographic information, knowledge assessment, and assessment of theory of planned behavior constructs, as well as a questionnaire for assessing consumption of food groups, fast food, and snacks. The data were collected at two stages (once at baseline and once three months after the intervention), and then the changes in knowledge, attitude, subjective norms, the perceived behavioral control, behavioral intention, and nutritional behavior were analyzed using descriptive and inferential statistical methods (t-test, Chi-square, Mann- Whitney U, and Wilcoxon tests; as well as one- way ANOVA, repeated measures ANOVA, and ANCOVA, respectively).

**Results:**

Statistically significant increases were observed in the mean scores of knowledge, attitude, subjective norms, the perceived behavioral control, behavioral intention, and nutritional behavior in the intervention group by passing three months from the intervention compared to the scores before the intervention (*P* < 0.001). However, these differences were not statistically significant in the control group (*P* > 0.05). Moreover, the mean scores of knowledge, attitude, subjective norms, perceived behavioral control, behavioral intention, and nutritional behavior had no statistically significant difference before the intervention between the two groups; however, this was statistically significant by passing three months from the intervention (*P* < 0.001).

**Conclusion:**

Considering the effect of an educational intervention program based on the theory of planned behavior on creating healthy nutritional behaviors in adolescents and young adults with polycystic ovary syndrome, it is recommended to use it in order to improve the nutritional health of them.

*Trial registration:* IRCT, IRCT20160224026756N6. Registered 18 Aug 2018, https://en.irct.ir/user/trial/32693/view

## Background

Polycystic ovary syndrome (PCOS) is the most common endocrine disorder in adolescents and adults [1], which is characterized by oligomenorrhea, amenorrheaacne, hyperandrogenism, hirsutism, hyperinsulinemia, and obesity [2]. Based on its diagnostic criteria, the prevalence rate of this disease among adolescent girls aged between 11 and 19 years old has been reported to be 1.8–15% [3]. In a study conducted on high school female students, the prevalence rate of clinical hyperandrogenism and oligomenorrhea was 19.9%; clinical hyperandrogenism and polycystic ovaries 30.8%; oligomenorrhea and polycystic ovaries 29.5%; and clinical hyperandrogenism, oligomenorrhea, and polycystic ovaries was 14.5% [4]. Currently, obesity is one of the main problems in industrial life and it is estimated that its prevalence in adolescents is 12–20% [5]. It appears that PCOS has increased in adolescents due to unhealthy nutritional behaviors in recent years [6]. According to previous studies, obesity is present in more than 50% of adolescent with PCOS. In addition, it was shown that obesity can exacerbate all metabolic complications and infertility in these people [7].

Unhealthy nutritional behaviors are known as important factors in the development of obesity during adolescence, which continues into adulthood [8]. Healthy nutritional behaviors and nutrition education play an important role in promoting the nutritional health of adolescents with PCOS. Thus, moving towards improving the nutritional awareness of people and proposing some strategies to improve their behaviors and lifestyle in terms of the individual and social components, have been widely considered in recent years (2016–2017) [9–11]. Abdolahian et al. in their meta- analysis concluded that lifestyle interventions such as diet and exercise, have the ability of improving some clinical, metabolic, and hormonal parameters in adolescents with PCOS [12]. In order to change a behavior, various theories and models have been proposed [13]. According to the theory of planned behavior (TPB), intention is the main determinant of behavior, and three factors of attitude, subjective norms, and the perceived behavioral control must be understood to affect a behavioral intention [14]. Mazloomy-Mahmoodabad et al. in their study showed that the TPB-based interventions seem to be effective on losing weight in obese and overweight adolescents, so it can be an appropriate pattern to plan for educational interventions [15]. Due to unhealthy nutritional behaviors as well as overweight/obesity, which are common among adolescents and adults with PCOS and may precipitate subsequent reproductive and metabolic disorders [16], the need for proposing an intervention based on the correction of unhealthy nutritional behaviors appears to be useful and cost-effective. Since no study has been conducted on evaluating the effect of educational interventions on nutritional behaviors of adolescents and adults with PCOS in Iran so far, so the present study aimed to determine the effect of an educational intervention program based on the TPB on nutritional behaviors of adolescents and young adults with PCOS.

## Methods

CONSORT guidelines were adhered for reporting this trial.

### Study design

This field trial study (IRCT20160224026756N6) was a part of a mixed-method study and conducted on the two groups (intervention and control) in 2018 [17]. In order to obtain an easier access to adolescents and young adults with PCOS from different cultural and social classes, the present study was performed in four gynecology clinics affiliated to Shiraz University of Medical Sciences, Iran. Based on the confidence interval of 95%, a test power of 80%, the standard deviation of 0.1 to 0.25 [18], a sample loss rate of 10%, and the equation used for sample size calculation $N=\frac{\left(Z_{1-{\displaystyle\frac\alpha2}+}Z_{1-\frac\beta2}\right)^2\left(S_1^2+S_2^2\right)}{\left(X_1-X_2\right)^2}$ a total of 36 people were estimated for each group.

In the current study, the inclusion criteria were as follows: (i) single overweight (with a body mass index (BMI) of 25 or more) or obese (with a body mass index of 30 or more) adolescents and young adults aged between 15 and 21 years old [19] with PCOS; (ii) diagnosis of PCOS by a gynecologist in terms of the diagnostic criteria [1]; (iii) without a history of any known psychiatric disorders under medical treatment; (iv) without a history of any known chronic disease such as diabetes, cardiovascular, and kidney disease; (v) without having any special diet prescribed by a doctor; vi) not participating in other clinical trials simultaneously; and (vii) participating in educational course sessions with a healthy peer. The exclusion criteria were the absence in more than 2 sessions of the educational classes, lack of willingness to continue cooperating in research, and using special diets during conducting this research.

### Procedures

**Fig. 1:**
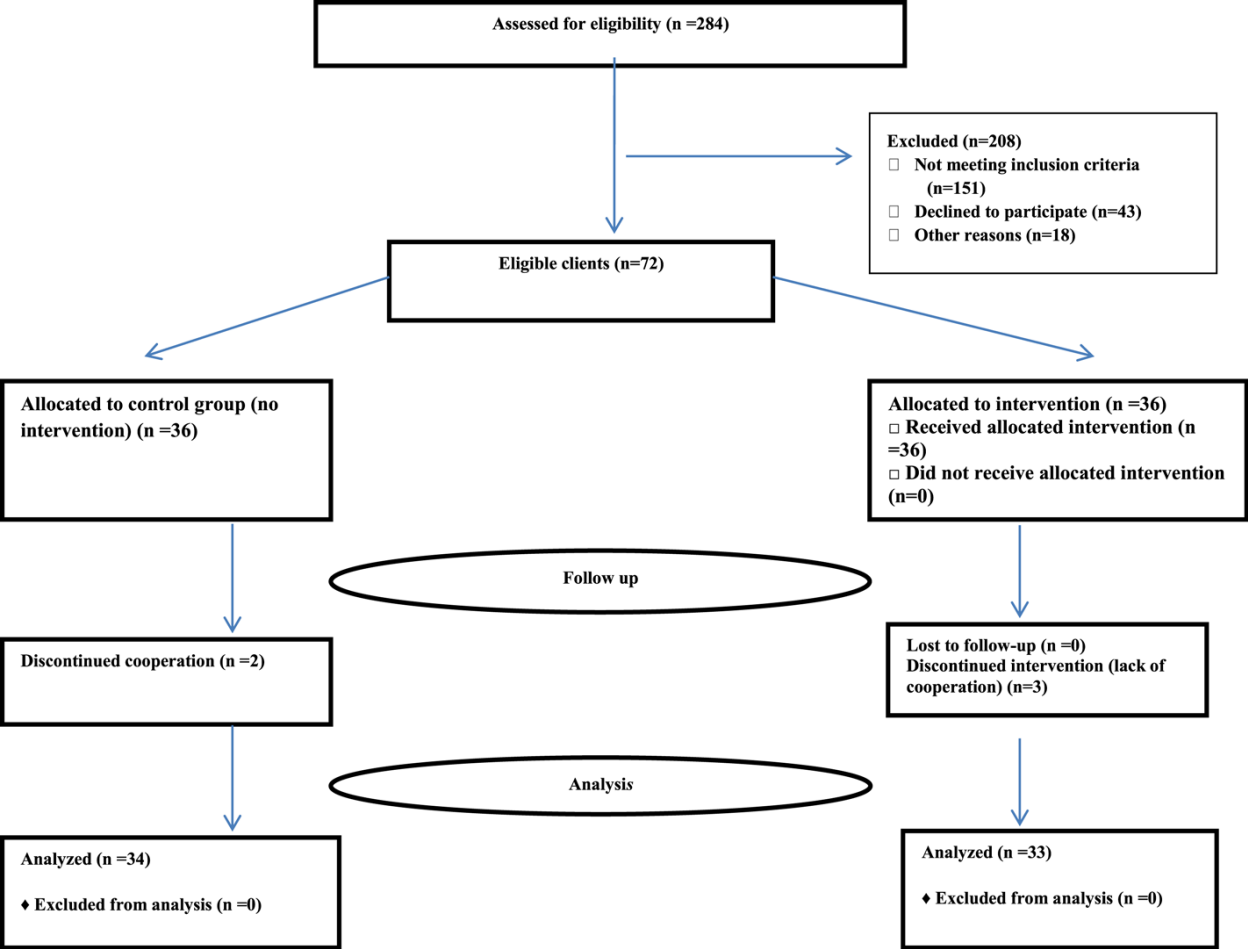
Trial flowchart

In this study, the sampling method used was convenience, so that the medical files of 284 adolescents and young adults with PCOS were evaluated and participants who met the inclusion criteria were selected in the gynecology clinics. Subsequently, after making phone calls to them, the time of each visit was settled. Among the 137 adolescents and young adults with PCOS referred to the four study clinics, 72 subjects were registered and after obtaining written consent, they were enrolled in the study (Fig. 1). In this study, 18 participants were selected from each clinic. The allocation of these people to the intervention and control groups was performed based on the order of their admission to the clinic. In each clinic, the first person was accepted and then assigned to the intervention group, and the second person was assigned to the control group, and by repeating this manner, the allocation process continued up to the end. In this study, after necessary coordination with the participants in the intervention group, the educational intervention program was performed based on the TPB in four sessions (60 to 90 min) during two weeks (two sessions per week). The researcher asked the participants to attend the training sessions at the patient education unit in one of the gynecology clinics with a suitable environment for training. At the end of the study, the participants' travel expenses were reimbursed and they were then appreciated by a gift. To improve the educational process during the training sessions, adolescents and young adults with PCOS (who were invited along with a same-sex healthy peer) were divided into three groups each one consisting of 12 participants. Peer attendance at the training sessions could help the participants understand why their peers want to change their nutritional behavior and support them in this regard [20]. The sessions included lectures, group discussions, questions and answers, role-playing, movies, brain storming, and practical display. Moreover, some strategies and activities were performed in the educational intervention program, which are presented in Table 1. In the present study, an educational session was also performed for the included participants’ mothers (without the presence of their daughters) to explain the principles of nutrition, healthy nutritional behaviors, PCOS and its side effects, and the effect of healthy nutrition on the treatment of PCOS, and finally an educational booklet was given to them. The participants in the control group received no training and they only completed the questionnaires at two stages. In this regard, a training booklet was given to them and also, if necessary, they were referred to a nutritionist after the end of the study. At this stage, by passing three months from the completion of the educational intervention, the researcher sent reminder messages once a week by forming a group in WhatsApp Messenger to follow the activities and nutritional behaviors of the participants included in the intervention group. Correspondingly, these messages were related to the topics and key points presented in the training sessions. Consequently, this provided a platform for questions and answers as well as the transfer of participants' experiences about their nutritional behaviors. Take advantage of this social media created a suitable platform for participants to communicate with the researcher easily if they need any further information.

**Table 1:** Strategies and activities used in educational intervention program

Construct	Strategy	Activity
Attitude	1-Strengthen the process of valuing and prioritizing healthy lifestyles and healthy nutritional behaviors (focus on the emotional component of attitude) 2-Convincing towards healthy lifestyle and nutritional behavior (strengthening cognitive attitude) 3-Clarify the main function of healthy nutritional behavior and the positive consequences of healthy nutritional behavior in improving PCOS 4-Group discussion 5-Share ideas	1-Provide comprehensive, accurate, evidence-based and realistic information about healthy nutrition and improving PCOS 2-Group discussion on the crucial role of weight loss and healthy nutritional behavior in improving PCOS 3-Group discussion on the problems and barriers to healthy nutrition in adolescents and young adults with PCOS
Subjective norms	1-Awareness of subjective norms 2-Role playing 3-Peer participation by presenting experiences and discussing each other's experiences 4-Feedback and encouragement to each other	1-Analysis of subjective norms 2-Discuss the role of important people (mothers, friends, and peers) about healthy nutritional behaviors 3-Discuss and provide solutions for forming affirmative group norms
Perceived behavioral control	1-Increased confidence on the feasibility of healthy nutritional behavior, reducing the barriers and complexities of healthy nutritional behavior 2-Overcoming the difficulties of performing the action	1-Discuss ways to overcome barriers to healthy nutritional behavior 2-Discuss the facilitators of healthy nutritional behavior, identify and reinforce them 3-Practice and implement healthy nutritional behaviors step by step 4-Provide opportunities to report direct experiences about healthy nutritional behaviors and provide verbal encouragement
Behavioral intention	1-Identify people's desires for healthy nutritional behaviors 2-Analyze the advantages and disadvantages of healthy nutritional behavior 3-Group decision and public commitment	1-Discuss and comment on the tendency of adolescents and young adults with PCOS to adopt healthy nutritional behaviors 2-Discussion to recall the short-term and long-term benefits of healthy nutritional behavior 3-Discussion to form a commitment and group decision

### Primary outcomes

In this study, the primary outcomes were knowledge, attitude, subjective norms, and perceived behavioral control about disease-related nutritional behaviors; intention for healthy nutritional behaviors; and participants' nutritional behaviors that were previously measured by the researcher-made questionnaires at two stages (once before the intervention and once three months after the intervention). The above-mentioned questionnaires contained demographic characteristics; knowledge about disease-related nutritional behaviors; attitude, subjective norms, and perceived behavioral control about the disease-related nutritional behaviors; intention for having healthy nutritional behaviors; and the consumption of food groups, fast food, and snacks. The demographic profile questionnaire was used to assess the participant’s age, occupation, and level of education, as well as parental employment status and educational level. The other questionnaires (used to measure knowledge, attitude, subjective norms, the perceived behavioral control, intention for healthy nutritional behaviors, and nutritional behaviors) were designed by studying the existing articles and resources based on the current research objectives [21, 22]. In the knowledge measurement about the disease-related nutritional behaviors, 11 questions (right, wrong, and I'm not sure) were used with a score of zero for not sure, or the wrong answer and score of one for the correct answer. Notably, the minimum knowledge score was zero and the maximum one was 11. Attitude about disease-related nutritional behaviors with 9 questions, subjective norms with 6 questions, perceived behavioral control with 7 questions, and intention for healthy nutritional behaviors with 8 questions were measured in a five-point Likert scale. The attitude scores ranged from 9 to 45, the subjective norms scores ranged from 6 to 30, the perceived behavioral control scores ranged from 7 to 35, and the intention for healthy nutritional behaviors scores ranged from 8 to 40.

The participants were asked to report the frequency of the consumption of fast food, sandwiches, snacks, and other food groups including bread and cereals, fruits and vegetables, milk and dairy products, legumes, nuts, and oily seeds (according to the specified daily share among the adolescent and young adults age groups [19]) during the past week. For designing the initial format of the questionnaire, the study by Keshani et al. [23] was used. Thereafter, using the opinion of nutritionists and by taking the nutritional needs of adolescents and young adults into account based on the food groups [19, 24, 25], changes were made in the questionnaire, which was finally designed with 11 items in the form of a 4-point Likert scale. In terms of nutritional behaviors, scoring for the consumption of fast food and snacks was "I never consume" as score 4, "sometimes I consume" (1–2 times a week,) as score 3, "I often consume" (3–4 times a week) as score 2 and "I always consume (equal to or more than 5 times a week)" as score 1. Regarding the food groups, scoring was done reversely. The range of nutritional behaviors was between 11 and 44. To validate the data collection tools, the content validity method was used and to determine the reliability of the questionnaires, the test–retest was used and Pearson correlation coefficient equal to 0.76 confirmed the reliability of the data collection tools.

### Statistical analysis

Data analysis was performed using the statistical package for the social sciences software 21.0 (SPSS version 21.0). Normality of distribution of the variables was investigated using the Kolmogorov–Smirnov test. The homogeneity of the study’s groups (about demographic characteristics) was assessed using chi-square and t-test. Additionally, the relationship between demographic characteristics and score of variables was examined using one- way ANOVA and t-test. For the purpose of this study, parametric and nonparametric tests were used. Accordingly, the parametric tests involved two assumptions as follows: one is that the populations for the dependent variable are normally distributed and the second is homogeneity of variance. Nonparametric tests were used for the analysis of the obtained data with a non-normal distribution. Moreover, statistical analysis was performed in the following two parts: intragroup and intergroup. Repeated measures ANOVA was also used to evaluate the intragroup relationship. To compare the mean score of variables and mean differences in scores of variables between the two study groups, the Mann–Whitney *U* test and ANCOVA were used. To compare the frequency of variables (food consumption) before and after the intervention in the intervention group, Wilcoxon test was used. The significance level for statistical tests was considered less than 0.05.

### Ethical considerations

Research ethics confirmation (ethical approval code: ethics code: IR.MUI.Rec.1395.3.885) was received from the Ethics Committee of Isfahan University of Medical Sciences. Written informed consent from the adolescent and her parents (in cases younger than 18 years old), anonymity, confidentiality, and the right of leaving the research at any time were preserved.

## Results

Among 36 participants in each group, 33 participants were included in the intervention group and 34 participants in the control group. Three participants in the intervention group were excluded due to the absence in the educational classes, and two participants in the control group were excluded due to treatment with a regimen. The results showed that there was no difference in demographic characteristics between the intervention and control groups (Table 2). Moreover, no statistically significant relationship was found between demographic characteristics (including educational level, Job, mother’s educational level, mother’s job, father’s educational level, and father’s Job) and mean scores of knowledge, attitude, subjective norms, the perceived behavioral control about the disease-related nutritional behaviors, intention for healthy nutritional behaviors, and nutritional behaviors (*P* > 0.05). However, there was a statistically significant relationship between age and the mean score of knowledge (*P* = 0.01).

**Table 2:** Comparison of demographic characteristics between intervention and control groups

Variable		Control group		Intervention group		Result of statistical test
		Number	%	Number	%	
Age (years)	Younger than 18	17	50	15	45.5	*t*=0.31
	18 and older	17	50	18	54.5	*P* = 0.760
Education	High school	17	50	17	51.5	*χ*^2^=1.60
	High School diploma	8	23.5	11	33.3	*P* = 0.510
	Associate degree	9	26.5	5	15.2	
Job	Student	23	67.6	25	75.7	*χ*^2^=3.41
	University student	8	23.5	3	9.1	*P* = 0.180
	Unemployed	1	2.9	3	9.1	
	Employed	2	6	2	6.1	
Mother’s education	High school	4	11.8	5	15.2	*χ*^2^=1.56
	High School diploma	15	44.1	12	36.4	*P* = 0.910
	Associate degree, B.S and Ph.D	15	44.1	16	48.5	
Father’s education	High school	10	29.4	9	27.3	*χ*^2^=0.14
	High School diploma	17	50	18	54.5	*P* = 0.930
	Associate degree, B.S and Ph.D	7	20.6	6	18.2	
Mother’s job	Stay-at-home mom	25	73.5	24	74.8	*χ*^2^=0.005
	Employed	9	26.5	9	27.2	*P* = 0.580
Father’s job	Self-employed	13	38.2	12	36.4	*χ*^2^=1.10
	Employee	13	38.2	10	30.3	*P* = 0.770
	Worker	5	14.7	8	24.2	
	Others	3	8.8	3	9.1	

### Primary outcomes

The results showed that the mean scores of knowledge, attitude, subjective norms, the perceived behavioral control about the disease-related nutritional behaviors, intention for healthy nutritional behaviors, and nutritional behaviors had statistically significant differences before and after the intervention in the intervention group, but no significant difference was observed in the control group (Table 3). As well, the results showed that the mean scores of knowledge, attitude, subjective norms, the perceived behavioral control about disease-related nutritional behaviors, intention for healthy nutritional behaviors, and nutritional behaviors had no statistically significant differences between the two groups before the intervention. But, the mean difference of scores of knowledge, attitude, subjective norms, the perceived behavioral control, behavioral intention, and nutritional behavior had a statistically significant difference between the two intervention and control groups (Table 4).

**Table 3:** Comparison of knowledge, theory of planned behavior constructs, and nutritional behavior before and after the intervention in each group

Group	Intervention group		Result of repeated measures ANOVA	Control group		Result of repeated measures ANOVA
Variable	Mean	SD	*P*-value	Mean	SD	*P*-value
Before the intervention	5.1	1.3	*P* < 0.001	4.7	0.5	*P* = 0.18
Knowledge	7.1	1.2		5	0.8	
After the intervention						
Before the intervention	26.1	2.1		25.9	3.5	
Attitude	30.9	2.9	*P* < 0.001	25.8	3.3	*P* = 0.59
After the intervention						
Before the intervention	20.2	1.6		19.9	1.7	
Subjective norms	22.7	1.6	*P* < 0.001	19.8	1.5	*P* = 0.43
After the intervention						
Before the intervention	22.8	2.3		22.2	3.5	
Perceived behavioral	26.6	2.2	*P* < 0.001	22.4	3.4	*P* = 0.87
control						
After the intervention						
Before the intervention	23.2	2.5		22.5	1.1	
Behavioral intention	28.3	1.1	*P* < 0.001	22.4	2	*P* = 0.45
After the intervention						
Before the intervention	20.6	1.6		20.9	1.5	
Nutritional behavior	23.8	1.4	*P* < 0.001	21	1.7	*P* = 0.14
After the intervention

**Table 4:** Comparison of knowledge, theory of planned behavior constructs, and nutritional behavior before and after the intervention between the two groups

Group	Intervention group	Control group	Result of Mann- Whitney test**	Result of ANCOVA***	
Variable	Mean ± SD	Mean ± SD		MD (95% CI)*	*P*-value
Before the intervention	5.1 ± 1.3	4.7 ± 0.5	*P* = 0.18	2.1 (1.54–2.54)	*P* < 0.001
Knowledge	7.1 ± 1.2	5.0 ± 0.8			
After the intervention					
Before the intervention	26.1 ± 2.1	25.9 ± 3.5	*P* = 0.93	5.1 (3.60–6.63)	*P* < 0.001
Attitude	30.9 ± 2.9	25.8 ± 3.3			
After the intervention					
Before the intervention	20.2 ± 1.6	19.9 ± 1.7	*P* = 0.18	2.9 (2.13–3.67)	*P* < 0.001
Subjective norms	22.7 ± 1.6	19.8 ± 1.5			
After the intervention					
Before the intervention	22.8 ± 2.3	22.2 ± 3.5	*P* = 0.72	4.2 (2.82–5.44)	*P* < 0.001
Perceived behavioral	26.6 ± 2.2	22.4 ± 3.4			
control					
After the intervention					
Before the intervention	23.2 ± 2.5	22.5 ± 1.1	*P* = 0.24	5.9 (4.75–7.03)	*P* < 0.001
Behavioral intention	28.3 ± 1.1	22.4 ± 2.0			
After the intervention					
Before the intervention	20.6 ± 1.6	20.9 ± 1.5	*P* = 0.42	2.8 (2.02–3.55)	*P* < 0.001
Nutritional behavior	23.8 ± 1.4	21.0 ±1.7			
After the intervention					

*Mean Difference (95% Confidence Interval)

**To compare the two groups before the intervention

***To compare the two groups after the intervention

The results of the present study showed that the frequency of the consumption of pizza, sandwiches, hamburgers, hot dogs, and all kinds of snacks by the participants in the intervention group decreased by passing three months from the start of the educational intervention compared to before the intervention and these differences were statistically significant (*P* < 0.001). In addition, the frequency of the consumption of food groups, including fruits and vegetables; milk and dairy products; and meat, seafood, legumes, and eggs increased in the participants of the intervention group by passing three months from the intervention compared to before the intervention, and these differences were statistically significant (*P* < 0.001) (Table 5).

**Table 5:** Comparing the frequency of consumption of food groups, fast food and snacks before and after the intervention in the intervention group

Frequency	Always (≥ 5 times a week)		Often (3–4 times a week)		Sometimes (1–2 times a week)		Never		Result of Wilcoxon test
Food	Before	After	Before	After	Before	After	Before	After	
	N (%)	N* (%)	N (%)	N (%)	N (%)	N (%)	N (%)	N (%)	
Pizza, sandwiches, hamburgers and hot dogs	6 (17.6)	1 (3)	18 (55.9)	6 (17.6)	9 (26.5)	24 (71.6)	0 (0)	2 (5.9)	*P* < 0.001
All kinds of snacks	8 (23.5)	2 (5.9)	18 (55.9)	13 (38.2)	7 (20.6)	18 (55.9)	0 (0)	0 (0)	*P* < 0.001
Fruits and vegetables	1 (3)	6 (17.6)	9 (26.5)	23 (70.6)	15 (46.9)	4 (11.9)	8 (23.5)	0 (0)	*P* < 0.001
Milk and dairy products	1 (3)	3 (9.1)	8 (23.5)	29 (87.9)	19 (58.3)	1 (3)	5 (15.2)	0 (0)	*P* < 0.001
Meat, sea food, legumes and eggs	1 (3)	2 (5.9)	4 (11.9)	7 (20.6)	18 (55.9)	19 (58.3)	10 (29.1)	5 (15.2)	*P* < 0.001

*Number

## Discussion

This study aimed to determine the effect of an educational intervention program based on the TPB on nutritional behaviors of adolescents and young adults with PCOS. According to the results, after the implementation of the educational intervention program, the participants, knowledge on disease-related nutritional behaviors more increased in the intervention group compared to the control group. In this regard, in Anderson et al.'s study, knowledge changes in the intervention group were found to be significantly higher than the control group, thus confirming the positive effect of educational intervention on improving students' nutritional knowledge [26]. It seems that in the present study, the use of educational strategies such as lecturing and role-playing has been effective on promoting the participants' knowledge on the disease-related nutritional behaviors in the studied age groups. Although knowledge alone is not enough to shape and change any behavior [27], it can pave the way for people to change their beliefs and facilitate the processes of accepting and adopting healthy nutritional behaviors to control and manage their disease.

In the present study, after the implementation of the educational intervention program, the participants, attitude about the disease-related nutritional behaviors more improved in the intervention group compared to the control group. Notably, attitude can affect behavioral intention and ultimately nutritional behaviors, and it has been mentioned in various studies such as the study by Martens et al. [28]. In the present study, it appears that the use of educational strategies and emphasis on the role of healthy nutritional behaviors and the consequences of these behaviors in PCOS management have strengthened the positive beliefs on nutritional behaviors, and finally, the participants’ attitude towards healthy nutritional behaviors improved.

In the present study, after implementing the educational intervention program, the participants’ subjective norms regarding the disease-related nutritional behaviors more improved in the intervention group compared to the control group. It appears that adolescents and young adults, understanding of the affirmative and supportive roles of their mothers and peers have been found to be effective on improving their subjective norms regarding healthy nutritional behaviors. Contrary to this finding, in the study by Brouwer & Mosack, they found that there was no statistically significant difference between the two groups of intervention and control in this study in terms of subjective norms regarding healthy nutritional behaviors and consumption of fruits and vegetables in adolescents [29]. In the present study, it seems that the participation of mothers and peers led the adolescents and young adults with PCOS to realize that important others support them and want to change their nutritional behaviors.

In the present study, after implementing the educational intervention program, the participants’ perceived behavioral control about the disease-related nutritional behaviors more increased in the intervention group compared to the control group. In many previous studies, the perceived behavioral control has been reported as one of the most important factors affecting both the adolescents' behavioral intentions and behaviors [30]. It is believed that increasing one's awareness and belief about having the ability to perform certain behaviors can be one of the factors affecting people's control beliefs [31]. In the present study, it appears that the acquisition of the necessary skills by the participants regarding the use of healthy nutritional behaviors (through interactive discussion, introducing successful patterns among peers, and practical exercises) has strengthened the ability of controlling beliefs related to applicable nutritional behaviors.

In the present study, after the implementation of the educational intervention program, the participants, intention for healthy nutritional behaviors more increased in the intervention group compared to the control group. As well, Grønhøj et al. in their study showed the effectiveness of educational intervention based on the TPB on improving adolescent’s nutritional intentions [23]. Additionally, Bashirian et al. in a survey study concluded attitude, subjective norms, and perceived behavior control as the most influential predictors of intention to abuse drugs [32]. In the present study, this finding showed the effectiveness of the educational intervention program on improving attitude, subjective norms, and perceived behavioral control in adolescents and young adults with PCOS that eventually led to the formation of intention for healthy nutritional behaviors.

In the present study, after implementing the educational intervention program, the participants, nutritional behavior more improved in the intervention group compared to the control group. In this regard, the participants in the intervention group reduced the consumption of foods such as pizza, sandwiches, hamburgers, hot dogs, and all kinds of snacks and also increased the consumption of fruits and vegetables; milk and dairy products; and meat, seafood, legumes, and eggs. As well, Mohammadi Zeidi et al. in their study showed that training based on the TPB can promote healthy nutritional behaviors, increase breakfast consumption, and reduce unhealthy snacks [33]. Based on the results of this study, it can be stated that age of female adolescent with PCOS could influence their knowledge. Given that during adolescence period, thinking style changes from concrete to hypothetical and abstract [19], it seems that older girls have more knowledge on changing nutritional behaviors to control their illness; therefore, they more intend to reduce their unhealthy nutritional behaviors.

### Practical implications

According to the obtained results, the application of the educational intervention program based on the TPB is effective on promoting the nutritional behaviors of adolescents and young adults with PCOS. Therefore, findings of the present study can be useful in designing and improving the educational programs available in the health system for educating adolescents and young adults with PCOS. Using this educational program can consequently empower adolescents and young adults with PCOS to adopt healthy nutritional behaviors and also to promote their nutritional health status and ultimately their reproductive health.

### Strengths and limitations

One of the strengths of this study was the implementation of educational intervention program based on the TPB for the adolescents and young adults with PCOS for the first time. One of the limitations of the present study was the assessment period of the educational program’s results, which was considered as three months after the intervention. However, in order to better assess the results of the educational intervention program, the follow-up period can be considered as longer in future studies. As well, in this study, the assessment of the final nutritional behavior was done based on the self-report of the participants, so it could have bias. In this regard, in future studies, a combination of self-reporting method, direct observation of behavior, and report by parents or teachers can be used if possible. In the present study, due to the convenience sampling and the possibility of selection bias, generalization of the results to a larger population of adolescents and young adults with PCOS may not be possible. Furthermore, since this study was non-blinded, this was another limitation that may have led to reporting bias. Also, in this field trial study, we faced a multiple testing problem which could have an impact on error rates, leading to inappropriate interpretation of the results.

## Conclusion

Based on the findings, the educational intervention program based on the TPB made appropriate changes in the nutritional behaviors of the adolescents and young adults with PCOS. Therefore, it is recommended to use this program to improve the nutritional health of these people, which can ultimately improve their reproductive health.

## Data Availability

The datasets generated and/or analysed during the current research are not publicly available as individual privacy could be compromised but are available from the corresponding author on reasonable request.

## Abbreviations

PCOS: Polycystic Ovary Syndrome
TPB: Theory of Planned Behavior
BMI: Body Mass Index
